# Editorial: Mechanisms and interventions for post-operative neurocognitive disorder and sleep disruptions

**DOI:** 10.3389/fneur.2025.1681931

**Published:** 2025-09-25

**Authors:** Bin Yang, Qi Chen, Zhaolan Hu

**Affiliations:** ^1^Department of Anesthesiology, The First Affiliated Hospital of Xiamen University, Xiamen, Fujian, China; ^2^Department of Anesthesiology, Chongqing University Cancer Hospital, Chongqing, China; ^3^Department of Anesthesiology, The Second Xiangya Hospital of Central South University, Changsha, Hunan, China

**Keywords:** neurocognitive disorder, sleep disruptions, post-operative, interventions, mechanism

## Introduction

Postoperative neurocognitive disorders (PNDs), encompassing postoperative delirium (POD) and postoperative cognitive dysfunction (POCD), represent a spectrum of cognitive impairments that occur following surgery, particularly in elderly populations. These conditions are associated with prolonged hospital stays, increased healthcare costs, and diminished quality of life. Sleep disturbances, often driven by disruptions to the circadian rhythm, are closely linked to PNDs, exacerbating cognitive decline through a vicious cycle of neuroinflammation and physiological stress. This Research Topic, Mechanisms and Interventions for Postoperative Neurocognitive Disorder and Sleep Disruptions, presents 17 cutting-edge studies exploring the underlying mechanisms and evaluating novel interventions. This review synthesizes these findings, categorizing the studies into those addressing mechanisms, interventions, or both. It also discusses their implications for clinical practice and future research.

## PNDs mechanisms and sleep disruptions

### Systemic and neuroinflammation factors

Neuroinflammation is a central mechanism driving PNDs. Gou et al. utilized transcriptomic analysis to identify differentially expressed genes (DEGs) such as COL18A1 and CXCL9 that interact with pro-inflammatory cytokines (e.g., IL-6, IL-1β) in elderly patients with POD. Their study employed correlation charts and immune infiltration plots to highlight the role of immune microenvironment interactions in neuroinflammation. Similarly, Liu Y. et al. developed a predictive model for postoperative pulmonary infections (PPI) in elderly orthopedic patients, identifying six clinical risk factors associated with PNDs. Their model, validated through calibration curves and decision curve analysis, underscores systemic inflammation as a key driver, despite limitations from retrospective data collection.

Qin et al. further elucidated the role of inflammation by demonstrating that elevated preoperative high-sensitivity C-reactive protein (Hs-CRP) levels significantly increase POD risk, particularly in older and female patients. Their findings position Hs-CRP as a potential biomarker for early risk stratification. Wei et al. identified preoperative biomarkers, including the neutrophil-to-lymphocyte ratio (NLR) and the systemic immune-inflammation index (SII) as predictors of pre-poststroke depression, which shares mechanistic overlap with PNDs, reinforcing the role of inflammatory pathways.

### Cognitive reserve and demographic factors

Xiang et al. investigated the influence of cognitive reserve and found that lower educational levels were associated with an increased risk of POD in patients undergoing abdominal surgery (odds ratio: 0.6, 95% CI: 0.8–0.9). These results suggest that cognitive reserve modulates neurocognitive outcomes, highlighting the need for preoperative cognitive assessments. A meta-analysis by Wu et al. revealed sex-specific vulnerabilities in neurological outcomes, with forest plots indicating that sex differences may influence PNDs risk, necessitating tailored intervention strategies.

### Circadian rhythm dysregulation

Ma et al. explored the interplay between postoperative pain, circadian rhythm disruptions, and neurocognitive outcomes. Their review highlights how pain-induced stress disrupts circadian rhythms, exacerbating PNDs through neuroinflammation and hormonal imbalances. These findings underscore the importance of effectively addressing pain management to mitigate sleep disturbances and cognitive decline.

## Interventions for PNDs and sleep disruptions

### Non-pharmacological interventions

Acupuncture has emerged as a promising non-pharmacological intervention for PNDs. A systematic review by Bu et al. demonstrated that acupuncture significantly reduced extubation time and stabilized hemodynamic parameters in postoperative settings, suggesting its efficacy in mitigating complications. Additionally, Wu et al. reported that electroacupuncture (EA) improved sleep duration and reduced the incidence of supraventricular tachycardia in patients undergoing thoracic surgery, likely by modulating autonomic nervous system activity.

Ultrasound-guided transversus abdominis plane (TAP) block also shows potential. A case report and literature review by Zhang et al. highlighted the ability of the TAP block to address anesthetic challenges in elderly patients with ovarian tumors and massive ascites, reducing postoperative neurocognitive complications by minimizing diaphragm elevation.

### Pharmacological and perioperative strategies

A systematic review by Wang Y. et al. suggested that remimazolam, a novel benzodiazepine, may alleviate POD, as assessed by MMSE scores and funnel plots. This highlights the potential of remimazolam as a pharmacological intervention. A clinical trial by Luo et al. demonstrated that tailored positive end-expiratory pressure (PEEP) in robot-assisted surgeries reduces pulmonary stress, potentially mitigating PNDs and sleep disruptions by improving respiratory function.

Zheng et al. evaluated the use of noninvasive ventilation post-extubation in ICU settings, finding that it may reduce pulmonary complications and support neurocognitive outcomes; however, data limitations warrant further investigation. Zhao et al. explored intratumoral injections of cisplatin and Endostar combined with cryotherapy/hyperthermia for malignant tracheal stenosis, suggesting that relieving airway obstruction may reduce surgical stress and support neurocognitive recovery.

### Tailored surgical and perioperative approaches

Wang Q. et al. compared the perioperative outcomes in 527 patients with and without pheochromocytoma, using odds ratios to assess the risk of complications. Their findings advocate for tailored surgical strategies to optimize perioperative conditions and reduce PNDs risk. A systematic review by Yang et al. emphasized the need for standardized guidelines for POD assessment, using a literature screening flowchart to ensure consistent diagnostic approaches, which are critical for effective management.

## Discussion

The studies in this Research Topic highlight the multifactorial nature of PNDs and sleep disruptions, which are driven by neuroinflammation, systemic inflammation, and circadian dysregulation. Interventions such as acupuncture, ultrasound-guided nerve blocks, and novel pharmacological agents like remimazolam show promise in mitigating these complications. However, challenges remain, including the need for prospective studies to overcome the limitations of retrospective data (e.g., Liu J. et al.) and the need to standardize diagnostic criteria (e.g., Yang et al.). Future research should focus on validating biomarkers such as Hs-CRP and NLR, refining predictive models, and exploring personalized interventions based on demographic factors such as education and gender.

## Conclusion

This Research Topic underscores the complex interplay between PNDs, sleep disturbances, and their underlying mechanisms, including neuroinflammation, systemic inflammation, and circadian misalignment. Innovative interventions, ranging from non-pharmacological approaches such as acupuncture and ultrasound-guided nerve blocks to pharmacological and perioperative strategies, offer promising avenues for improving postoperative outcomes. By addressing these challenges through rigorous research and tailored interventions, we can enhance recovery and quality of life for surgical patients, particularly those in vulnerable populations.

